# Retraction: The role of NF-κB and miRNA in oral cancer and cancer stem cells with or without HPV16 infection

**DOI:** 10.1371/journal.pone.0242853

**Published:** 2020-11-18

**Authors:** The PLOS ONE Editors

Following the publication of this article [1], concerns were raised regarding the results presented in Figs 1, 4, 5, 6, and 7. Specifically,

- In Fig 1B, the FACS results presented for the Oral tumor biopsy (HPV+ve) Verapamil (-ve) appear more similar to the FACS results for the Oral tumor biopsy (HPV+ve) Verapamil (+ve) than would be expected from independent FACS samples. The authors have indicated that inadvertent errors have been made during the assembly of the figure panels, and that the raw underlying data to support the FACS results presented in Fig 1B are no longer available.
- Similarities were observed between the following FACS results presented in this article [1] and FACS results depicting different experimental conditions in an article previously published in Frontiers in Oncology [2]. The authors have indicated that the FACS data presented in the latter article is incorrect, but they have not provided raw underlying data to support the FACS results presented in this article:
  ○ The UPCI:SCC131 (HPV-ve) Verapamil(-ve) panel of Fig 1A [1] appears similar to the UPCI:SCC84 (HPV-ve) Verapamil (-ve) panel of Fig 1 [2].
  ○ The Oral tumor biopsy (HV-ve) Verapamil (-ve) panel of Fig 1B [1] appears similar to the UPCI:SCC131 (HPV-ve) Verapamil (-ve) panel of Fig 1 [2].
  ○ The Oral tumor biopsy (HV-ve) Verapamil (+ve) panel of Fig 1B [1] appears similar to the UPCI:SCC131 (HPV-ve) Verapamil (+ve) of Fig 1 [2].
- Similarities have been detected between results presented in the western blot panels presented in Fig 4B and Fig 5A. The authors indicated that these similarities are the result of inadvertent errors that occurred during figure preparation:
  ○ The UD-SCC2 Oct 4 panel of Fig 4B appears similar to the UPCI-SCC 84 (HPV-) p52 and the UPCI-SCC 84 (HPV-) Rel-B panels of Fig 5A. In addition, the second (NSP) and third (SP) lanes of the forementioned panels appear similar to the second (NSP) and third (SP) lane of the UD SCC-2 (HPV 16+) p52 panel of Fig 5A.
  ○ The UPCI:SCC131 Sox-2 panel of Fig 4B appears similar to the UD SCC-2 (HPV 16+) c-Rel panel of Fig 5A and the UPCI-SCC 84 (HPV-) c-Rel panel of Fig 5A.
  ○ In Fig 5A, the second (NSP) lane of the UD SCC-2 (HPV 16+) Rel-B panel appears similar to the first (Parental) lane of the UPCI-SCC 84 (HPV-) p65 panel.
- Irregularities have been detected in the background signal of results presented in Figs 4B, 5A, 6A, 7A, and 7C. The authors indicated that the irregularities are the result of gel splicing conducted to ensure the panels demonstrated pertinent results only:
  ○ A vertical irregularity was detected between the first and the second lane of the UPCI:SCC131 Oct 4 panel of Fig 4B.
  ○ Vertical irregularities were detected between the first and the second lanes of the UD SCC-2 (HPV 16+) p50, p52, and Rel-B panels of Fig 5A.
  ○ Horizontal and vertical irregularities were detected around the second lane of the UPCI-SCC 131 (HPV-) p52 panel and the second lane of the UPCI-SCC 84 (HPV-) p65 panel of Fig 5A.
  ○ A horizontal irregularity was detected between the second and the third lane of the HPV-ve OSCC biopsy p50 panel of Fig 6A.
  ○ A vertical irregularity was detected directly below the bands presented in the (ii) panel of Fig 7A.
  ○ Discrepancies were detected in the background surrounding the results presented in the second and third lane of the NSP panel of Fig 7C (ii).

The underlying raw FACS data and the individual level data for the various graphs presented in this article were not made publicly available, although the Data Availability statement states that all relevant data are within the manuscript and its Supporting Information files. The authors provided underlying data for some but not all the blots presented in this article, however the data received by the journal were not sufficient to resolve the concerns summarised above.

In light of the concerns affecting multiple figures that question the integrity of the reported results, the *PLOS ONE* Editors retract this article.

BCD agrees with the retraction decision. NB, MY, and DM either did not respond directly or could not be reached.
